# THE BABY-LED WEANING METHOD (BLW) IN THE CONTEXT OF COMPLEMENTARY FEEDING: A REVIEW

**DOI:** 10.1590/1984-0462/;2018;36;3;00001

**Published:** 2018

**Authors:** Ana Letícia Andries e Arantes, Felipe Silva Neves, Angélica Atala Lombelo Campos, Michele Pereira

**Affiliations:** a Universidade Federal de Juiz de Fora, Juiz de Fora, MG, Brasil.

**Keywords:** Child, Weaning, Infant nutrition, Baby-led weaning, Criança, Desmame, Nutrição infantil, Baby-led weaning

## Abstract

**Objective:**

To review the scientific findings on the baby-led weaning method (BLW) in the context of complementary feeding.

**Data sources:**

Two independent examiners searched the Medical Literature Analysis and Retrieval System Online (MEDLINE)/PubMed database in August 2016. No time-period was defined for the publication dates. The following descriptors were used: “baby-led weaning” OR “baby-led” OR “BLW”. Inclusion criteria were: original studies that were available in English, and which addressed the BLW method. Exclusion criteria were: references in other languages, opinion articles and literature reviews, editorials and publications that did not elaborate on the intended subject. Of the 97 references identified, 13 were included in the descriptive synthesis.

**Data synthesis:**

The BLW group of babies, when compared to the traditional eating group, were less prone to being overweight, less demanding of food, and ate the same foods as the family. The number of choking episodes did not differ between groups. Mothers who opted for the implementation of BLW had higher levels of schooling, held managerial positions at work, and were more likely to have breastfed until the sixth month of the child’s life. Concerns were raised about messes made during meals, wasting food, and choking, but most of the mothers recommended adopting the method. Health professionals were hesitant to indicate this method.

**Conclusions:**

BLW was recommended by mothers who followed the method with their own children. However, concerns have been reported, which, coupled with professionals’ fears about the inability of infants to self-feed, reflect a lack of knowledge about the method.

## RESUMO

**Objetivo:**

Revisar as constatações científicas a respeito do método *baby-led weaning* (BLW) no âmbito da alimentação complementar.

**Fontes de dados:**

Buscas conduzidas na base de dados Sistema Online de Busca e Análise de Literatura Médica (MEDLINE)/PubMed em agosto de 2016 por dois examinadores independentes, sem delimitação de período. Foram utilizados os descritores: “*baby-led weaning*” *OR* “*baby-led*” *OR* “BLW”. Critérios de inclusão: estudos originais, disponibilizados em inglês, que abordaram o tema do método BLW. Critérios de exclusão: referências em outros idiomas, artigos de opinião e de revisão da literatura, editoriais e publicações que não discorreram sobre o assunto pretendido. Das 97 referências identificadas, 13 foram incluídas na síntese descritiva.

**Síntese dos dados:**

Os bebês adeptos ao BLW, quando comparados aos do grupo em conduta alimentar tradicional, foram menos propensos ao excesso de peso, menos exigentes em relação ao alimento e consumiam os mesmos alimentos da família. Os episódios de engasgo não diferiram entre os grupos. As mães que optaram pela implementação do BLW exibiram mais escolaridade, ocupavam um cargo gerencial no trabalho e apresentaram maior probabilidade de terem amamentado até o sexto mês. Foram mencionados preocupações com bagunça nas refeições, desperdício de comida e engasgo/asfixia, mas a maioria recomendava a adoção do método. Os profissionais da saúde demonstraram receio em indicá-lo.

**Conclusões:**

O BLW foi sugerido pelas mães que o seguiram com seus filhos, todavia relataram-se preocupações, que, somadas ao receio dos profissionais acerca da capacidade dos bebês de se autoalimentarem, refletem escassez de conhecimento sobre o método.

## INTRODUCTION

The phase in a baby’s life when exclusive breastfeeding stops and complementary feeding begins is replete with numerous questions. The World Health Organization (WHO) advocates for exclusive breastfeeding for children up to six months of age, with no additional water, tea or any type of food.^1^ It is only after this six-month period that supplementary feeding is recommended.^1^ ^,^ ^2^

The introduction of foods that have a pasty consistency is a traditionally widespread practice, but it has also been subject to debate. As such, the baby-led weaning method (BLW) - named by Gill Rapley, author of the book *Baby led weaning: helping your baby to love good food* - suggests that infants as young as sixth months old have the ability to determine their own food intake. Therefore, the babies that demonstrate adequate growth and development are able to begin to consume pieces of food, without the need for substantial changes in food consistencies.^3^ ^,^ ^4^

According to the conceptual framework, this method offers opportunities for children to choose:

1. when to start their meals;
2. what they will eat. Food is chosen among the healthy options offered by their caregivers;
3. the rhythm of the meals;
4. the amount of food that will be ingested at each meal.^4^

As such, the caregivers serve as food intermediaries because they make the babies’ food available and they provide a pleasant environment, so the babies can exercise their motor skills and try a wide variety of foods, thus getting to know the act of eating in its entirety.^3^ ^,^ ^4^

In short, the act of offering pieces of food represents the role of a facilitator for infant self-feeding, as this encouragement is fundamental for the method.^4^ However, there is still no consensus about the safety of this practice, not even in relation to the potential reflections in eating behavior and in growth and development. Furthermore, the references are scarce and the are no publications found in the Portuguese language.

As such, this study proposes a review of the scientific findings present in the literature with respect to the BLW method in the context of complementary feeding and, in this way, it establishes a comprehensive body of knowledge on the topic.

## METHOD

This study is an integrative literature review that was formulated by means of ordered procedures, with the intention of critically identifying, selecting, and analyzing references that deal with the topic.

With the objective of helping give structure to the present study, a protocol was adopted with the following questions:^5^ ^,^ ^6^ recognizing the subject and establishing a guiding question; defining inclusion and exclusion criteria for the references; searching in the electronic literature; selecting and categorizing the identified references by evaluating the titles and abstracts; performing an eligibility stage by evaluating the full texts; critical reading of the texts to determine what information would be taken away; and finally, a descriptive synthesis of the content.

### Databases, electronic searches, and critical readings

The methodological criteria and a flow chart were adapted based on the recommendation of the Preferred Reporting Items for Systematic Reviews and Meta-Analyse (PRISMA), in accordance with the descriptions contained in the following topics: ^7^

1. databases: by means of preliminary searches conducted during the “recognition of the subject” phase, it was confirmed that the references of interest were scare and there were duplicates of them. Because of this, the Medical Literature Analysis and Retrieval System Online (MEDLINE)/PubMed database, which is a notable source of scientific health information, was chosen as the sole database.
2. subject descriptors and Boolean operator: “*baby-led weaning*” *OR* “*baby-led*” *OR* “BLW”*.* According to the *Medical Subject Headings* (MeSH), these terminologies are not in the controlled vocabulary, however it was necessary to use them, given that the references of interest mentioned at least one of the terms in the title and/or keywords. Adding on other possible descriptors like, “*weaning*”, “*complementary feeding*” and “*infant nutrition*” would not be advantageous in the screening, but would problematize the selection and eligibility stages;
3. inclusion criteria: original studies available in the English language from quantitative or qualitative research that approach the BLW method from an infant feeding behavior or growth/development perspective in addition to the knowledge and/or conduct of mothers and health professionals;
4. exclusion criteria: references in languages other than English, literature review articles, opinion articles, editorials, and publications that did not specifically deal with the present subject;
5. electronic searches: performed by two independent examiners in August of 2016. No time-period was defined. The disagreements during the selection and eligibility stages were solved beforehand with a consensus. Given the persistent controversies, a third examiner gave their opinion;
6. critical readings: the eligible studies were submitted to independent and paired critical readings by means of *checklists* from the *Critical Appraisal Skills Program* (CASP).^8^ ^,^ ^9^ The following criteria were considered: first, clarity in identifying the objectives; second, appropriate methodology (the recruitment of participants and data collection); third, the relationship between researcher and participants in regard to ethics; and fourth, analysis of the data, results and research contributions.

### Establishing the references

**Figure 1:**
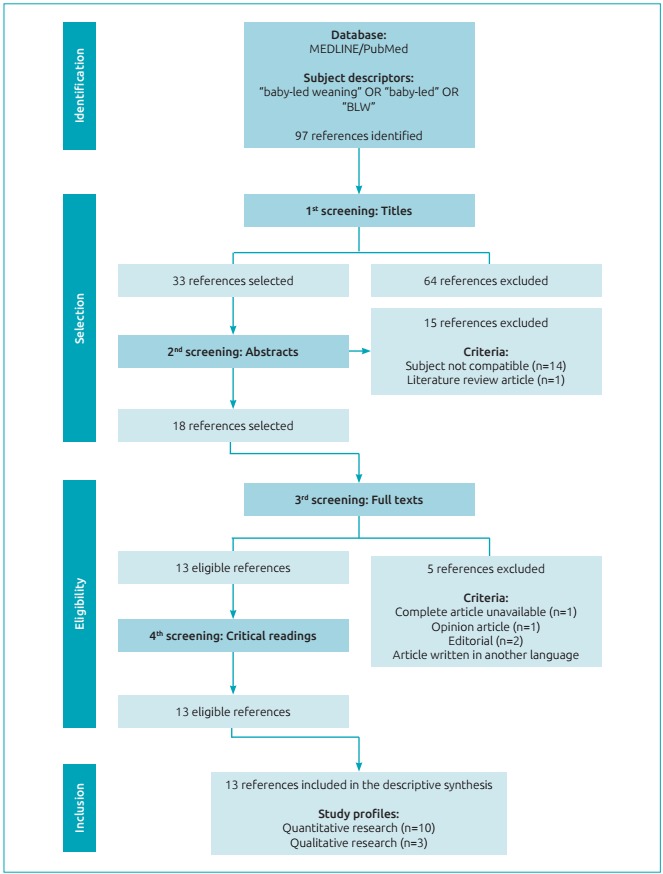
Flow chart of the stages: identification, selection, eligibility, and inclusion of the references.

Figure 1 demonstrates the flow chart showing the identification, selection, eligibility, and reference inclusion stages. The examiners evaluated the titles of 97 publications obtained in the database, and then they discarded 64 files. Afterward, 30 abstracts were verified, and 15 references were excluded, among which 14 approached complementary feeding through a perspective other than BLW, and one consisted of a literature review. It is important to clarify that, of the 33 publications admitted into the screenings because of their titles, three did not have abstracts, and because of this, were forwarded directly to the eligibility stage.

In the end, thorough evaluations of the full texts were performed, however one of the articles was not available, one was an opinion article, two were editorials, and one was written in German. The 13 references that remained were submitted to critical readings and all of them fulfilled at least 80% of the questions observed in the checklists, and they did not result in new exclusions.

## RESULTS

The descriptive synthesis was composed of 13 references^10^ ^,^ ^11^ ^,^ ^12^ ^,^ ^13^ ^,^ ^14^ ^,^ ^15^ ^,^ ^16^ ^,^ ^17^ ^,^ ^18^ ^,^ ^19^ ^,^ ^20^ ^,^ ^21^ ^,^ ^22^ - 10 came from quantitative studies^10^ ^,^ ^11^ ^,^ ^12^ ^,^ ^13^ ^,^ ^14^ ^,^ ^17^ ^,^ ^18^ ^,^ ^20^ ^,^ ^21^ ^,^ ^22^ and three had qualitative methodologies^15^ ^,^ ^16^ ^,^ ^19^ -, and their dates of publication were between 2011 and 2016. With regard to the designs of the quantitative studies, seven were cross-sectional,^10^ ^,^ ^12^ ^,^ ^14^ ^,^ ^17^ ^,^ ^20^ ^,^ ^21^ ^,^ ^22^ one was a case-control study ^13^ and two were cohort studies.^11^ ^,^ ^18^ Data collection from the qualitative studies was performed by means of semi-structured interviews.^15^ ^,^ ^16^ ^,^ ^19^

Table 1 shows a brief description of all the references with the following items: author9s0 (year), title, location of the study, objectives, design and sample. Table 2 contains the 13 citations that deal with BLW from an infant feeding behavior and/or growth/development perspective. Eight of them came from the United Kingdom^10^ ^,^ ^11^ ^,^ ^12^ ^,^ ^13^ ^,^ ^16^ ^,^ ^18^ ^,^ ^19^ ^,^ ^22^ one from the United Kingdom/United States,^14^ three from New Zealand^15^ ^,^ ^17^ ^,^ ^20^ and one from Canada.^21^ Table 3 includes the 10 references that approach BLW from a maternal perspective. Six of them were from the United Kingdom^10^ ^,^ ^12^ ^,^ ^16^ ^,^ ^18^ ^,^ ^19^ ^,^ ^22^ one was from the United Kingdom/United States,^14^ two were from New Zealand^15^ ^,^ ^17^ and one was from Canada.^21^ Only two citations also dealt with BLW in regard to health professionals. One of them was from New Zealand,^15^ and the other was from Canada.^21^

**Table 1:** A brief description of the references included in the systematic review.

Author(s) (year)*	Title	Location of the study	Objectives	Design and sample
Brown & Lee^10^ (2011)	A descriptive study investigating the use and nature of baby-led weaning in a UK sample of mothers	Swansea, United Kingdom	To characterize a sample of mothers who adhered to BLW as a strategy for feeding their children, as well as to describe attitudes and behaviors associated with the practice of this method	Cross-sectional study 655 mothers of babies 6 to 12 months old
Wright et al*.* ^11^ (2011)	Is baby-led weaning feasible? When do babies ﬁrst reach out for and eat ﬁnger foods?	United Kingdom	Describe the age range in which children first reached out to pick up food, relating this to self-feeding, aspects of child development, and socioeconomic status	Cohort study 510 mothers of babies born in 1999 and 2000
Brown & Lee^12^ (2011)	Maternal control of child feeding during the weaning period: differences between mothers following a baby-led or standard weaning approach	United Kingdom	To compare the feeding profile between BLW-adhering infants and infants following traditional feeding behavior	Cross-sectional study 604 mothers of babies 6 to 12 months old
Townsend & Pitchford^13^ (2012)	Baby knows best? The impact of weaning style on food preferences and body mass index in early childhood in a case-controlled sample	Nottingham, United Kingdom	To compare the dietary profile and the BMI of children who adhered to BLW and others who followed traditional eating habits	Case-control study 155 mothers of children between 20 and 78 months old
Rowan & Harris^14^ (2012)	Baby-led weaning and the family diet. A pilot study	United States and United Kingdom	To investigate whether the implementation of the BLW method affected the mother’s diet and whether the same family foods were offered to the babies	Cross-sectional study 10 mothers of babies approximately 6 months old
Cameron et al*.* ^15^ (2012)	Healthcare professionals’ and mothers’ knowledge of, attitudes to and experiences with, baby-led weaning: a content analysis study	Dunedin, New Zealand	To evaluate the knowledge, attitudes and experiences of health professionals, as well as mothers of babies following BLW, about this method	Qualitative study with semi-structured interviews 31 health professionals and 20 mothers of babies 8 to 24 months old that adhered to BLW
Brown & Lee^16^ (2013)	An exploration of experiences of mothers following a baby-led weaning style: developmental readiness for complementary foods	United Kingdom	To examine the attitudes, beliefs and behaviors adopted by mothers who have opted for using BLW to feed their children	Qualitative study with semi-structured interviews 36 mothers of babies between 12 and 18 months old
Cameron et al*.* ^17^ (2013)	Parent-led or baby-led? Associations between complementary feeding practices and health-related behaviours in a survey of New Zealand families	New Zealand	To compare feeding practices and health behavior between BLW adherents and others who have followed traditional feeding behavior	Cross-sectional study 199 mothers of babies between 6 and 12 months old
Brown & Lee^18^ (2015)	Early influences on child satiety-responsiveness: the role of weaning style	United Kingdom	To compare feeding behavior between babies weaned with the BLW method and those following traditional feeding behavior	Cohort study 298 mothers of babies between 18 and 24 months old
Arden & Abbott^19^ (2015)	Experiences of baby-led weaning: trust, control and renegotiation	United Kingdom	To investigate the experiences reported by mothers who chose to practice BLW in order to understand the benefits, challenges and beliefs about this method	Qualitative study with semi-structured interview 15 mothers of babies between 9 and 15 months old
Morison et al*.* ^20^ (2016)	How different are baby-led weaning and conventional complementary feeding? A cross-sectional study of infants aged 6-8 months	New Zealand	To compare the feeding profile between BLW-adhering infants and infants undergoing traditional feeding behavior	Cross-sectional study 51 mothers of babies between 6 and 8 months old
D’Andrea et al*.* ^21^ (2016)	Baby-led weaning: a preliminary investigation	Canada	To investigate the practice of BLW with regard to the knowledge and perceptions of mothers and health professionals about the method	Cross-sectional study 33 health professionals and 65 mothers
Brown^22^ (2016)	Differences in eating behaviour, well-being and personality between mothers following baby-led vs. traditional weaning styles	United Kingdom	To compare the demographic and socioeconomic profiles of mothers who used BLW and others who chose traditional feeding behavior	Cross-sectional study 604 mothers of babies between 6 and 12 months old

*Studies are ordered chronologically; BLW: baby-led weaning; BMI: body mass index.

**Table 2:** The baby-led weaning method from an infant feeding behavior and/or growth/development perspective.

Author(s) (year)*	Main results
Brown & Lee^10^ (2011)	the duration of exclusive breastfeeding was substantially higher among mothers who followed the BLW method; the caregivers who opted for the method were more likely to offer fruits or vegetables as the babies’ first foods; most of the children being cared for at an institution because their mothers returned to work, were fed with potatoes / pastes; the method was associated with greater participation in family meals.
Wright et al*.* ^11^ (2011)	56% (n=340) of the babies had stretched out their hands to grab food before their sixth month, of which 27% (n = 92) were considered to be incapable of self-feeding; in general, for many 8-month-old children, self-feeding was still not a routine part of their meals, and they were described as being fully fed by the caregiver.
Brown & Lee^12^ (2011)	mothers who adhered to the BLW method perceived that their children weighed more at 6 months old
Townsend & Pitchford^13^ (2012)	carbohydrates were the preferred food category for infants who adhered to the method, while the group following traditional eating habits preferred sweet foods; children following traditional behavior had a higher BMI, and were more susceptible to being overweight.
Rowan & Harris^14^ (2012)	the infants consumed, on average, 57% of the same foods ingested by the mothers, with minimum and maximum similarities of 44 and 86%, respectively.
Cameron et al*.* ^15^ (2012)	90% (n = 18) of the mothers started to practice the BLW method by their children’s fifth or sixth month of life; the first foods commonly offered were fruits and vegetables; 80% (n = 16) of the mothers reported that their children shared meals with one or more family members; there were reports of offering potatoes / mashed potatoes and the use of spoons, however this was only occasionally, when the infants were sick or appeared to be too fatigued to self-feed; mothers who cited episodes of choking said that the children dealt with the problem by themselves, expelling the food through coughing.
Brown & Lee^16^ (2013)	the babies usually participate in family meals; in general, the mothers described that the method stimulated the consumption of *in natura* foods.
Cameron et al*.* ^17^ (2013)	babies adhering to the BLW method were more likely to consume the same foods eaten by the family and were less likely to have received industrialized food; there was no difference between the groups adhering to the method and those following traditional feeding behavior with regard to choking episodes.
Brown & Lee^18^ (2015)	the duration of breastfeeding did not differ between BLW groups and traditional behavior groups, but mothers who followed the method were more likely to have started breastfeeding at birth; infants who adhered to the method were characterized as less food-demanding and more sensitive to being full; in general, children exhibited eutrophy, but those in traditional management weighed more.
Arden & Abbott^19^ (2015)	many mothers who followed the BLW reported using food as a toy early on in the implementation of the method.
Morison et al*.* ^20^ (2016)	the duration of exclusive breastfeeding was substantially higher among mothers who had adhered to the BLW. Moreover, children following traditional behavior consumed more infant formulas; there was no difference in terms of energy consumption, but the children who followed the method ate more fat (total and saturated) and lower amounts of iron, zinc and vitamin B12.
D’Andrea et al*.* ^21^ (2016)	the mothers started the BLW method around the fifth or eighth month of life of their children; the first foods commonly offered were fruits and vegetables. infants routinely participated in family meals.
Brown^22^ (2016)	the average age at the start of food intake was lower for the traditional group (19 weeks) than for the BLW group (24 weeks).

*Studies are ordered chronologically; BLW: baby-led weaning; BMI: body mass index.

**Table 3:** The baby-led weaning method from a maternal perspective.

Author(s) (year)*	Main results
Brown & Lee^10^ (2011)	58.2% (n = 384) of mothers considered themselves to be well-informed about the practice of BLW; the degree of knowledge about the method was inversely proportional to the caregivers’ use of a spoon and to the offering of potatoes / pastes; those who adhered to the BLW were married, had more schooling, and held a managerial position at work - or their partners had these characteristics; traditional food practitioners more often resorted to the support of health professionals, seeking clarification on complementary feeding. They also reported more anxiety about whether their children’s nutritional intake is appropriate, more concern about making messes during meals, and also felt more insecure about their babies’ chewing abilities.
Brown & Lee^12^ (2011)	mothers who adhered to the BLW had higher levels of education, were employed, and were more likely to hold a managerial position and not return to work; cited less concerns about the baby’s weight, food restrictions and pressure to eat.
Rowan & Harris^14^ (2012)	three months after the introduction of the method, the mothers did not show modifications in their diet.
Cameron et al*.* ^15^ (2012)	the BLW method was considered to be more convenient and less stressful for the introduction of food; mothers believed that the method helped their children to develop healthy eating behaviors; choking was not a concern, as mothers considered it to be natural for when children try new foods; the mess made during meals was highlighted as the main disadvantage of the method, however all mothers would recommend it to other mothers.
Brown & Lee^16^ (2013)	mothers considered the BLW method to be simple and convenient; meals were seen as easier and less stressful; in general, they reported that they exercised little control with regard to the amount of food ingested by the infants; the mess made at meals and food waste resulting from the practice of the method were considered challenges; they expressed concern with regard to the risk of choking, which decreased over time.
Cameron et al*.* ^17^ (2013)	all of the families who adhered to the BLW would recommend it, but more than half of them would advise that the practice be completed in conjunction with traditional complementary feeding; 46% (n = 65) of mothers who adhered to traditional eating habits, said that if they had another child, they would be willing to try BLW; the main reasons for not opting for the method were: choking; fear of lack of motor ability to guide self-feeding; and uncertainty about the adequacy of the amount of food ingested at each meal, in addition to the fact that traditional behavior had worked well in the past.
Brown & Lee^18^ (2015)	mothers who adhered to the BLW method reported less concern about the baby’s weight, pressure to eat, and food restrictions. In addition, they spent less time watching over the children at mealtimes.
Arden & Abbott^19^ (2015)	mothers who adhered to BLW reported less anxiety during meals; those who followed the method reported a high degree of confidence in the babies’ ability to choose the timing, type, and amount of food to eat; some expressed fears about nutritional support for the infants, as well as concerns about the mess made during meals.
D’Andrea et al*.* ^21^ (2016)	ch0king was the most cited concern with regard BLW, though it decreased over time; a large portion of the mothers had been introduced to the method through friends or online sources; more than 80% believed that the method would have promoted healthy eating behaviors, and improved children’s fine motor skills and oral development.
Brown^22^ (2016)	mothers who adhered to BLW had higher levels of schooling and were more likely to be in managerial positions; adherents to traditional eating habits had higher scores for anxiety and obsessive-compulsive disorder.

*Studies are ordered chronologically; BLW: baby-led weaning.

## DISCUSSION

### The baby-led weaning method from the infant feeding behavior and/or the growth/development perspective 

Brow and Lee^10^ were pioneers who formally characterized BLW in a study with 655 mothers of babies between 6 and 12 months old, who were residents of Swansea County in the United Kingdom. The researchers covered information about weaning and meal-time experience during the introduction of foods. Among the results, it stands out that the duration of exclusive breast feeding was substantially larger among the mothers who followed the method, a fact that is also reported in other investigations.^14^ ^,^ ^15^ ^,^ ^16^ ^,^ ^17^ ^,^ ^20^ ^,^ ^21^ In most of the cases, ingestion of the complementary foods began around the sixth month of life, and was thus is accordance with international principles.

In the long run, breastfeeding protects children against infections, dental malocclusion, excess weight, and diabetes.^23^ ^,^ ^24^ ^,^ ^25^ ^,^ ^26^ The world-wide prevalence of excess weight and obesity stands out that in the last two decades as it has acquired epidemiological characteristics, affecting the child and youth population in an alarming way and positioning this issue among the serious public health obstacles to be faced in the 21st century.^26^ ^,^ ^27^ ^,^ ^28^ As a result, the burden of chronic noncommunicable diseases exhibits increasing ratios in various countries, including developing ones, which have been historically marked by malnutrition.^29^ ^,^ ^30^ For these reasons, early behaviors - such as adequate breastfeeding - have been reiterated as a way to avoid harmful outcomes in adulthood.^23^ ^,^ ^26^ ^,^ ^29^

Confirming the findings from Brown and Lee’s exploratory research,^10^ subsequent studies demonstrated that babies who are adept to BLW were more likely to consume the same foods as the family and to share the same meal times.^14^ ^,^ ^16^ ^,^ ^17^ ^,^ ^21^ Participation in the familial context is of extreme importance, because imitation is one of the pillars of infant learning. The literature demonstrates the pertinence of the learning process in the formation of feeding behaviors, and that stimuli can last throughout childhood/adolescence and even into adulthood.^31^ ^,^ ^32^ ^,^ ^33^ Furthermore, there is a positive relationship between eating with the family and interacting with family members.^33^ Thus, the presence of the child in the same environment as the rest of the family while they are eating meals - in conjunction with healthy foods - is of great importance in aiding the implantation/continuation of the method.

In this context, Rowan and Harris^14^ investigated the probable compatibility between the foods ingested by the children and their families. After three months, starting from the introduction of the BLW, the babies consumed, on average, 57% of the same foods as the mothers, with a minimum and maximum similarity of 44 and 86%, respectively. Curiously, the participant with the least equivalent diet also consumed the family foods, but in separate occasions throughout the day. For example, the child would eat dinner from what the mother ate during lunch. This type of routine sharing possibly intensifies adherence to the method, because the caregivers save time on chores that are specifically for the preparation and feeding of meals to the baby, which makes the whole process less exhausting.

The foods that are commonly offered to the children in the beginning of the BLW were fresh fruit and vegetables and no industrialized products.^10^ ^,^ ^15^ ^,^ ^21^ In accordance with D’Andrea et al*.*,^21^ animal proteins made up the second most reported group, and included red meat, poultry, and fish. The foods were *in natura* or softened, and were made available in the form of strips or other manageable size bites.^10^ ^,^ ^14^ ^,^ ^15^ ^,^ ^21^

In contrast, Morisson et al*.* ^20^ affirmed that the babies that were adept to BLW had ingested high levels of fat and less iron, zinc, and vitamin B12. No differences were found between the energetic values consumed by the children from the method and traditional behavior. Despite this, in both groups, 45 and 76% received, respectively, sweetened foods and foods rich in sodium.

Rowan and Harris^14^ also found that, in children following the BLW method, even with a smaller proportion of rice, bread, crackers, cookies, yogurt, cheese, eggs, butter, soups and pasta, it is a common finding that frequent ingestion of sugary foods, cookies, and fat is associated with excessive weight gain and its consequences.^26^ ^,^ ^29^

Townsend and Pitchford,^13^ in a case-control study that evaluated 155 babies between 20 and 78 months old, observed that the ones that adhered to BLW preferred carbohydrates, while the group following traditional food behaviors had a preference for sweet foods. It was also found that babies that followed the method had a lower body mass index (BMI), and were more closely classified in the appropriate ranges. The children that followed the traditional behaviors had larger BMIs, and were more susceptible to being overweight.

Brow and Lee,^18^ in another study with babies between 18 and 24 months old, observed, that after one year of following the method, the BLW group was less demanding in relation to feeding, more sensitive to being full, and less prone to being overweight. The authors reiterated that the practice of the method provided a protecting environment that minimized the risk of obesity, something that is justified by the practice of eating healthier foods.

With regard to the occurrence of choking episodes, Cameron et al*.*,^17^ in an online study with 199 caregivers, found that no difference was detected between the BLW groups and the traditional behavior group. It should be highlighted that a large portion of the mothers were worried about suffocation from choking,^16^ ^,^ ^17^ ^,^ ^21^ however this is uncommon with BLW and can be confused with the gag reflex (or a vomit reflex), especially because the reflex comes from the back region of the mouth, at the base of the tongue. As such, the improperly chewed food returns to the back portion of the oral cavity before being swallowed. Next, either it will be spit out, or it will be chewed and swallowed.^31^

D’Andrea et al*.* ^21^ reported that only three children (4.6%) had experienced some choking incident while the method was being conducted. In their comments, the caregivers recognized the differences between choking and suffocation, but suggested that first aid training would be useful for the caregivers who practice BLW. In the study of Cameron et al*.*,^15^ the mothers that cited the occurrence of choking said that the child dealt with the problem on their own and got rid of the food through coughing.

### The baby-led weaning method from a maternal perspective

With regard to the motivations for implementing the BLW method, the mothers studied by Cameron et al*.* ^15^ and D’Andrea et al*.* ^21^ reported that the method “made sense”, “seemed logical”, and “was natural”. Most of them recommended it because they believed that it was a process that facilitated health eating habits,^15^ ^,^ ^17^ ^,^ ^21^ and that contributed to refining fine motor skills and oral development of the babies .^21^ The textures of the foods helped with sensorial perception and generated benefits related to orofacial growth. Because of this, a diet with a high consistency positively impacts the chewing function.^34^ ^,^ ^35^

The caregivers that adhered to the BLW, in comparison with those that practiced the traditional behavior methods cited:

1. little control over the amount of food ingested by the children, which was not always seen as something positive;^16^ ^,^ ^19^
2. significantly lower levels of concern with the weight of the babies, with the pressure to eat and with food restrictions;^12^ ^,^ ^18^
3. less time spent watching the children eat during meals;^12^ ^,^ ^18^ ^,^ ^19^
4. less anxiety;^19^ ^,^ ^22^
5. high confidence levels with regard to the babies’ capacity to regulate the duration of meals and choose the type and quantity of foods;^19^
6. less stress during the meals, as the method was more simple and convenient.^15^ ^,^ ^16^

Additionally, successive concerns were noted with regard to the ability of the babies to guide their own feeding, in addition to the uncertainty with the amount of food ingested and the nutritional input.^10^ ^,^ ^16^ ^,^ ^17^ ^,^ ^19^ The quantity and frequency in which the food is offered should be based on how much the child accepts, which varies according to individual necessities, the amount of breastmilk ingested, and the density of the complementary foods.^4^ ^,^ ^36^ The implementation of the BLW also requires signs of developmental dexterity, including postural balance to sit with little or no assistance, as well as stability to reach, grasp, and bring food to the mouth.^4^ ^,^ ^11^ ^,^ ^31^ These aptitudes manifest themselves when the child is around six months old,^17^ ^,^ ^36^ thus corroborating the recommendations of the WHO, which emphasizes exclusive breastfeeding up to the sixth month of life. Only after this period does it indicate the offering of other foods.^1^ ^,^ ^2^

It is emphasized that sudden weaning is not advisable, since in the beginning of BLW, a large part of the energetic and micronutrient necessities will still be provided by breastfeeding. Nevertheless, this process should happen gradually, according to the control signs shown by the baby.^4^ ^,^ ^31^

Wright et al*.* ^11^ analyzed motor development with regard to children’s self-feeding in the cohort in The Gateshead Millennium Baby Study. The researchers obtained the following findings:

1. among the caregivers that wrote in a diary about the 5 five times that their children consumed solid foods, 40.0% offered finger food (food that the baby can grab) before six months of age;
2. 56.0% of the children had held out their hands to hold on to food before their sixth month of life. Upon turning 8 months, 36.0% received finger food only once a day. And 27.0% were considered uncapable of feeding themselves;
3. of the total number of babies that had contact with finger food, 9.6% received them at least once a day when they reached 8 months;
4. 35.0% of the 8-month-old children were described as fully fed by the caregivers, as they were unable to do it themselves;
5. in summary, for many 8-month-old children, self-feeding is still not a routine at their meals.

It can be inferred that even though the babies demonstrated interest and readiness to feed themselves by their sixth month of life, the opportunities given to them by their caregivers were still insufficient. More opportunities would naturally lead to a greater proficiency in chewing and swolloing.^11^

Over time, the mothers that had mentioned concerns about choking episodes became more confident.^16^ ^,^ ^21^ The mess and waste of food that come from the practice of BLW were considered the greatest challenges.^15^ ^,^ ^16^ ^,^ ^19^

A great number of the caregivers had been introduced to the method by groups of parents, friends, or online sources*.* ^10^ ^,^ ^15^ ^,^ ^21^ In general, those that adhered to the BLW method were married,^10^ had higher levels of schooling, were in managerial positions at their work^10^ ^,^ ^12^ ^,^ ^22^ - or their respective spouses had these characteristics^10^ - and were more likely to not have returned to work during the period in which they introduced food to their children.^12^ Two investigations did not find any differences with regard to maternal age, marital status, or income.^12^ ^,^ ^22^

It was noted that those who adhered to traditional behavior used the support of health professionals more often in order to clarify uncertainties about the complementary diet.^10^ ^,^ ^15^

### The baby-led weaning method from the perspective of health professionals

Cameron et al*.*, ^15^ interviewed 31 health professionals in Dunedin, New England, and found that less than half (41.9%) were aware of the method. Furthermore, a large portion of them who participated in the research had not seen BLW in action, and, because of this, they firmly resisted understanding the child’s ability to coordinate the chewing/swallowing of pieces of food. Nevertheless, some advantages were explained with regard to the use of the method, for example:

- sharing meals as a family;
- incentivizing healthy eating habits;
- stimulating oral development through chewing;
- less stress for the care-givers during meals, given that the process is entirely managed by the baby’s rhythm.

D’Andrea et al*.*, ^21^ in a survey of 33 Canadian professionals, noted that 81.8% were aware of the BLW method and had heard about this conduct through other health professionals, patients or in training. More than 80% of the respondents believed that the method could promote the progress of fine motor skills and oral development in children. Even as mentioned in the previous section of this article (“The baby-led weaning method from a maternal perspective”), the same study revealed that a large number of mothers had been informed about the method through online sources, a fact that demonstrates the lack of mention of the BLW method among professionals in pediatrics.

Despite considering the approach to be beneficial, the majority did not feel completely convinced to recommend it, especially because of the concern regarding the risk of suffocation. Furthermore, it is believed that the practice of BLW may negatively affect caloric intake and iron intake.^15^ ^,^ ^21^

The apprehension shown by health professionals - which impacts the scarcity of recommendations of the method - results from the lack of theoretic-practical knowledge. Additionally, it is emphasized that the WHO, seen as the highest reference for decision making, continues to cautiously wait for more clinical evidence before taking an official position, something that without a doubt makes the adherence to BLM more difficult.

### Limitations of the studies reviewed

In general, the studies reviewed offered relevant contributions to the understanding of BLW in the context of complementary feeding. Nonetheless, the following limitations stand out:

1. the lack of standardized criteria for delimiting the proportion of passive dietary practices used in BLW results in methodological differences in the allocation of participants between the groups of the method and those of traditional behavior;
2. the information regarding maternal breastfeeding and complementary feeding can be influenced by memory, when the data is obtained in a retrospective way;
3. the predominance of studies with a cross-sectional design cannot guarantee a cause and effect relationship for the observed associations. In fact, the studies with small sample sizes and no probabilities have inferential limitations that are even larger;
4. it should be noted that all studies available in the literature evaluated exclusively the populations of Europe and North America, which restricts the extrapolation of certain findings to developing countries, since a number of sociocultural determinants are implicated in the practice of the method;
5. the use of the online environment as a way of recruiting and applying questionnaires compromises the internal and external validity of the results. Probably, the caregivers chosen through self-selection were more involved in the process of introducing food to the children. In addition, it stands out that this methodological procedure tends to choose samples with higher levels of schooling and income, a fact that justifies, in parts, the high concentration of participants with a high educational level and those that are part of the middle class.

In conclusion, the BLW was recommended by mothers who followed the method with their children. However, they reported concerns about the messes made at meal times, the wasting of food and the possibility of choking. These issues, coupled with the health professionals’ fears about babies’ inability to feed themselves, reflect the scarcity of recommendations and encouragement for the implementation of the method.

There were no differences in the proportion of children that choked between those who adhered to BLW and those that followed traditional feeding behavior. The method was associated with a longer duration of exclusive breastfeeding, child participation in family meals, greater self-regulation of being full, and less work for feeding the babies.

It is hoped that the present revision will broaden the knowledge on feeding methods and incite new investigations, given that the lack of bibliographical materials on this subject provides a wide field for scientific research.
